# An Intimate Relationship between ROS and Insulin Signalling: Implications for Antioxidant Treatment of Fatty Liver Disease

**DOI:** 10.1155/2014/519153

**Published:** 2014-02-12

**Authors:** Aurèle Besse-Patin, Jennifer L. Estall

**Affiliations:** ^1^Division of Cardiovascular and Metabolic Diseases, Institut de Recherches Cliniques de Montreal 110, Avenue des Pins Ouest, Montreal, QC, Canada H2W 1R7; ^2^Molecular Biology Department, University of Montreal, Montreal, QC, Canada H3C 3J7

## Abstract

Oxidative stress damages multiple cellular components including DNA, lipids, and proteins and has been linked to pathological alterations in nonalcoholic fatty liver disease (NAFLD). Reactive oxygen species (ROS) emission, resulting from nutrient overload and mitochondrial dysfunction, is thought to be a principal mediator in NAFLD progression, particularly toward the development of hepatic insulin resistance. In the context of insulin signalling, ROS has a dual role, as both a facilitator and inhibitor of the insulin signalling cascade. ROS mediate these effects through redox modifications of cysteine residues affecting phosphatase enzyme activity, stress-sensitive kinases, and metabolic sensors. This review highlights the intricate relationship between redox-sensitive proteins and insulin signalling in the context of fatty liver disease, and to a larger extent, the importance of reactive oxygen species as primary signalling molecules in metabolically active cells.

In humans, excessive storage of triglycerides in the liver, resulting from various etiologies including chronic over nutrition and physical inactivity, potentiates the development of nonalcoholic fatty liver disease (NAFLD). The first stage of the disease is simple steatosis, characterized by triglyceride deposits within lipid droplets in hepatocytes. Simple steatosis can progress to nonalcoholic steatohepatitis (NASH) following hepatocyte injury (hepatocyte ballooning, cell death), inflammation, and fibrosis. 20–40% of the population of industrialized countries have high levels of fat in their livers due to diet, sedentary lifestyle, and poor health [1–3]. Our understanding of the pathogenesis of nonalcoholic fatty liver disease (NAFLD) is limited by the difficulty of early detection and diagnosis [4]. Poor liver health affects many organ systems and impacts multiple arms of the metabolic syndrome, including cardiovascular health, insulin sensitivity, and circulating lipid levels [5, 6]. Half of obese humans (adults and children) have fatty livers and with the steadily increasing incidence of obesity, fatty liver disease will likely become a significant burden on our health care system [7]. NAFLD and NASH are also strongly correlated with insulin resistance, a major risk factor for type 2 diabetes [3]. Interestingly, the presence of liver fat is a better predictor of type 2 diabetes risk than obesity or BMI [8, 9]. In addition, NASH patients have a significantly higher risk of developing liver cancer (hepatocellular carcinoma) [10].

There is convincing evidence for a central role of mitochondrial dysfunction in the pathophysiology of NAFLD/NASH [11–18]. Mitochondrial dysfunction alters lipid metabolism, increases oxidative stress, and promotes proinflammatory cytokine production [11, 16]. Inflammatory cytokine signalling and stellate cell activation result in fibrosis and endoplasmic reticulum stress, stimulating apoptosis and necrosis [19, 20]. Altered oxidative phosphorylation and increased reactive oxygen species (ROS) levels are reported in patients with NASH [13–17] and are associated with structural abnormalities in liver mitochondria that appear swollen, rounded, and have decreased cristae density [16, 21]. Accordingly, decreases in the mitochondrial electron transport chain (ETC) complex activity and ATP synthase are reported in NAFLD patients [15]. Several studies in experimental animal models and humans indicate a strong association between the severity of NAFLD/NASH and degree of mitochondrial dysfunction and oxidative stress [12, 15, 22–24]. Increased serum oxidative markers (thioredoxin, oxidized LDL, thiobarbituric acid-reactive substances, malondialdehyde) are also observed in patients [22, 25]. Deficient antioxidant defenses are a major factor promoting oxidative stress and decreased coenzyme Q10, CuZn-superoxide dismutase, catalase activity, glutathione, and glutathione S-transferase correlate with the severity of liver disease [26, 27].

Oxidative stress occurs when there is an imbalance of pro-oxidant (ROS) formation and reduced antioxidant defenses [28]. ROS are free radicals derived from oxygen and include singlet oxygen (O^•^), superoxide anion (O^−^), and hydroxyl (HO^•^ or HO^−^) radicals. ROS are highly reactive, resulting in a short life and limited diffusion radius (e.g., super- oxide anion has a half-life of 10^−6^ s) [29, 30]. ROS are formed through radical leakage from enzymes such as nicotinamide adenine dinucleotide phosphate (NADPH) oxidase [31], cyclooxygenases [32], lipoxygenases [33], and the mitochondrial ETC [34]. The NOX family of NADPH oxidase enzymes are membrane-bound electron carriers, which use NADPH as electron donor and oxygen as acceptor. NADPH oxidases localize to cellular membrane compartments via targeting proteins, facilitating hydrogen peroxide (H_2_O_2_) production within spatially confined areas [31, 35]. There are various molecular mechanisms within a cell designed to counteract overproduction of ROS. Superoxide anion is dismutated by mitochondrial manganese superoxide dismutase (MnSOD) into H_2_O_2_, which is more chemically stable and has a potentially wider diffusion range. It is then detoxified into water by mitochondrial glutathione peroxidase-1 (GPx) [29, 36]. Mitochondrial catalase also has a detoxifying effect against overproduction of hydrogen peroxide [37].

Mitochondria are a principal source of cellular ROS due to electron leak along the ETC [37]. Oxidative reactions in the mitochondria (*β*-oxidation, Krebs' cycle) generate reduced cofactors (NADH and FADH_2_), which are then oxidized (NAD^+^ and FAD). Electrons formed by oxidation of these reduced cofactors are carried through the redox complexes of the respiratory chain (complexes I, III, and IV) to the final electron acceptor, molecular oxygen [37]. Mitochondrial ROS generation is governed by the redox state of the respiratory chain [38–40]. Electron transfer through the mitochondrial respiratory chain generates an electrochemical gradient and the energy of this gradient is used to generate adenosine triphosphate (ATP) by ATP synthase. Mitochondrial ROS generation can be modulated by bioenergetics substrates such as uncouplers, free fatty acids, or adenosine diphosphate (ADP) [34, 41, 42]. Mitochondria continually exposed to high levels of ROS can suffer deleterious consequences, such as oxidative damage to ETC complexes, mtDNA, or lipids [21], leading to mitochondrial dysfunction.

A master regulator of mitochondrial biogenesis and function is the transcriptional coactivator peroxisome proliferator-activated receptor-*γ* coactivator-1*α* (PGC-1*α*) [43, 44]. PGC-1*α* coordinates the transcriptional activity of several nuclear transcription factors such as nuclear respiratory factors 1 and 2 (NRF-1 and -2) and transactivates genes involved in the respiratory chain, mitochondrial import machinery, and transcription factors of mtDNA (such as the mtDNA transcription factor A (TFAM)). Decreased mitochondrial biogenesis associated with impaired biological activity of PGC-1*α* or reductions in TFAM has been observed in fatty livers [45–47]. Moreover, chronic liver-specific deletion of PGC-1*α*, or its homologue PGC-1*β*, in mice leads to hepatic steatosis and insulin resistance [48, 49]. PGC-1*α* also regulates the induction of antioxidant defenses including SODs, catalase, and GPx [50] and increases expression of the metabolic sensor NAD^+^-dependent deacetylase sirtuin 3 (Sirt3), a key regulator of the mitochondrial antioxidant system [51]. Deficient Sirt3 activity in livers predisposes mice to NASH [52]. In addition, nuclear Sirtuin 1 (Sirt1) deacetylates PGC-1*α* to regulate mitochondrial biogenesis and function [53]. Sirt1 is decreased in a rat model of NAFLD [54] and Sirt1 hepatic deficiency leads to oxidative damage and insulin resistance [55]. Thus, there is a strong mechanistic link between deficiencies of proteins controlling mitochondrial biogenesis, function, and antioxidant capacity and the development of fatty liver disease, stimulating great interest in targeting these pathways for new therapeutics [56].

While there is a compelling correlation between mitochondrial dysfunction and fatty liver disease, the molecular pathways directly linking altered mitochondrial function, insulin resistance, and inflammation are still unclear. While increased ROS remains an attractive theory, there is still much debate and little direct evidence on whether accumulated ROS due to mitochondrial dysfunction is a significant causative factor in NAFLD/NASH, in particular hepatic insulin resistance. Houstis and colleagues [57] show that ROS production precedes insulin resistance in cultured 3T3-L1 adipocytes and ROS scavenging rescues insulin sensitivity, providing evidence toward ROS as a primary cause of insulin resistance. To address whether mitochondrial ROS contributes to insulin resistance *in vivo*, Anderson and colleagues administered a potent small molecule antioxidant peptide, SS31, that specifically targets mitochondria [58, 59]. They show that coadministration of SS31 to rats consuming a high-fat diet for six weeks reduces mitochondrial H_2_O_2_ emission by 50%, normalizes the mitochondrial H_2_O_2_-emitting potential, and prevents the development of insulin resistance [58, 60]. Moreover, palmitate-induced hepatic insulin resistance is dependant on the generation of mitochondrial ROS [61], demonstrating that the negative effect of ROS on insulin signalling is a phenomenon shared by a variety of metabolic tissues. Other approaches to target mitochondria with antioxidants, such as transgenic mice overexpressing mitochondrial peroxiredoxin 3 [62] or targeted mitochondrial overexpression of catalase [63], have established mitochondrial H_2_O_2_ as a primary signal linking metabolic imbalance to insulin resistance.

Conversely, some argue that insulin resistance, resulting from nutrient overload and mitochondrial exhaustion, is the initiating event leading to mitochondrial dysfunction and increased ROS in metabolic disease [64]. However, insulin receptor deficiency, at least in muscle, does not generate oxidative stress [65] and most studies using environmental and nutritional factors to induce insulin resistance, such as a high-fat diet, report increased ROS and oxidative damage prior to the development of insulin resistance [66–68]. Interestingly, mice with whole body deficiency of GPx1, a key enzyme in ROS detoxification, fail to become obese mice on a high-fat diet, do not develop hepatic steatosis, and have improved hepatic insulin signalling [69]. While this suggests increased ROS does not necessarily lead to hepatic insulin resistance and fatty liver disease, global GPx1 deletion and the general failure of these mice to gain fat mass makes it difficult to isolate direct effects of GPx1 loss on liver metabolism. Further investigation using a conditional knockout approach will help clarify the role of GPx1, if any, in fatty liver disease. It is still not clear whether increased ROS is a major player in decreased insulin signalling in fatty liver disease. Well-designed studies using tissue-specific and inducible genetic models of insulin resistance (e.g., insulin receptor or insulin receptor substrates proteins knockouts) and more advanced tools that can detect changes in ROS production *in vivo* are needed to decipher the precise temporal link between insulin resistance and ROS.

Evidence linking increased ROS to insulin resistance is strong, yet it is still not well understood how alterations in hormone signalling are explained by variations in ROS concentration. Irreversible oxidative damage to key signalling mediators has been proposed; however, the system is further complicated by the fact that ROS are also essential players in many hormone-regulated cellular processes. H_2_O_2_ is widely accepted as a crucial signalling molecule [70, 71]. Generation of H_2_O_2_ at concentrations that are not thought to promote oxidative stress or damage is involved in the regulation of redox signalling pathways [71]. Of all cysteine residues within the proteome, it is estimated that more than 10% are redox-sensitive [71]. The sulphur atom within cysteine can exist as a reduced thiol (SH) or in different oxidized states, such as thiolate anion (S^−^), sulfenate (SO^−^), disulfide (S-S) sulfinate (SO_2_^−^), or sulfonate (SO_3_^−^) [22]. Alterations in the redox state of these sulphur atoms induce changes in protein conformation affecting enzyme activity, protein interaction, trafficking, degradation, and transcription factor binding to DNA [71]. In fact, the intracellular redox circuit is a master regulator of phosphorylation/dephosphorylation events in the cell due to the presence of redox-sensitive cysteine residues within nearly all classes of protein phosphatase enzymes [72]. Thus, phosphatase activity can be reduced in response to an oxidative shift in the redox environment. For example, protein tyrosine phosphatases (PTPs) are deactivated by oxidation of a conserved redox-sensitive cysteine residue within their catalytic sites [73]. The phosphoprotein family of Ser/Thr phosphatases also appear susceptible to oxidative deactivation [74, 75]. Protein phosphatase 2A (PP2A) and protein phosphatase 1 (PP1), which account for the majority of all Ser/Thr phosphatase activity [76], are sensitive to oxidative inactivation due to the presence of a conserved CXXC motif within their catalytic domains [74, 77].

Under resting conditions, the redox environment is in a reduced state and phosphatase activity exceeds kinase activity by 10-fold [78]. This generates an intracellular “phosphatase tone” preventing inappropriate phosphorylation events *in vivo *[75]. H_2_O_2_ emission leads to deactivation of key phosphatases, potentiating kinase activity and altering signal propagation in the cell. Imbalances between pro-oxidants and antioxidants that provoke phosphatase inactivation have been linked to disease development. For example, oxidative changes in phosphatase enzymes, such as MAP kinase phosphatase (MKP-1), phosphatase and tensin homolog (PTEN), and mitochondrial matrix targeted PP2C (PP2Cm) are linked to aging [79], cancer [80], and apoptosis [81]. Kinase and phosphatase activity play an integral role in propagation and regulation of the insulin response in cells.

The insulin signalling cascade begins with insulin binding to the insulin receptor at the cell surface, activating intrinsic tyrosine kinase activity (Figure 1). Insulin receptor substrate (IRS) docking proteins become phosphorylated, leading to the recruitment and activation of phosphoinositide 3-kinase (PI3K). A major substrate for PI3K is the membrane lipid phosphatidylinositol-4,5-bisphosphate (PIP_2_), which is phosphorylated to produce phosphatidylinositol-3,4,5-trisphosphate (PIP_3_). Increases in PIP_3_ attract pleckstrin homology domain-containing signalling proteins, such as the Ser/Thr kinases phosphoinositide-dependent protein kinase-1 (PDK1) and protein kinase B (PKB/Akt). Phosphorylated PDK1 activates the PKB/Akt kinase, which potentiates glycogenesis through phosphorylation of glycogen synthase kinase 3, stimulates fatty acid synthesis via activation of ATP citrate lyase, and inhibits gluconeogenesis by inhibitory phosphorylation of Forkhead box protein O1. PKB/AKT also promotes mTORC1 signalling to regulate protein synthesis. To amplify the signal, insulin binding initiates deactivation of phosphatases such as PTP1B, PTEN, and SH2 domain-containing phosphatase (SHP2) [82–84]. Inactivation of these phosphatases due to an oxidative shift in the redox environment at the level of the plasma membrane potentiates downstream kinase signalling [82, 83]. The source of the insulin-induced oxidative shift at the plasma membrane appears to be extracellular H_2_O_2_ generated by NOX complexes [85, 86] (Figure 1). Consistently, exposure to H_2_O_2_ markedly reduces Ser/Thr phosphatase activity in skeletal muscle [75] and hepatocytes [84]. Furthermore, this mechanism may be independent of insulin binding to its receptor. Adiponectin, known to increase insulin sensitivity, acts through small GTPase Rac1 activation and 5-lipooxygenase stimulation. It creates an ROS burst that deactivates PTP1B and increases insulin signalling [87]. In addition, receptor tyrosine kinase activation leads to the transient phosphorylation and inactivation of membrane-associated peroxiredoxin 1, a major H_2_O_2_-detoxifying enzyme [88]. Thus, insulin-stimulated tyrosine kinase and NADPH oxidase activation coordinate generation of an oxidized environment localized along the plasma membrane to inhibit phosphatase activity and facilitate insulin signalling. Once insulin levels decline, NADPH oxidase activity decreases and the local redox environment at the plasma membrane returns to a reduced state due to activity of the antioxidant systems (catalase, peroxiredoxins), restoring phosphatase activity and resetting the insulin signalling cascade at a basal state. Thus, insulin signalling requires ROS to be effective and it seems paradoxical that evidence points to elevated ROS as a primary cause of decreased insulin signalling and insulin resistance [57, 58]. However, there is growing evidence that altered activities of redox-sensitive phosphatases enzymes are implicated in the detrimental effects of ROS on the insulin signal pathway.

Several studies show that activation of stress-sensitive Ser/Thr kinases causes inhibitory phosphorylation of insulin receptor and IRS1/2. An oxidized environment due to ROS accumulation inhibits phosphatase activity, and thus promotes stress-kinase activity, providing a potential mechanism for ROS-induced insulin resistance [89]. Among these Ser/Thr kinases, p70-S6 kinase 1 (S6K1), c-Jun N-terminal kinase-1 (JNK1), extracellular signal regulated kinase-1 (ERK1), and inhibitor of NF-*κ*B kinase *β* (IKK*β*) have all been implicated in insulin resistance [89]. Furthermore, mice deficient for JNK1 [90], ERK1 [91], S6K1 [92], and IKK*β* [93] are protected from diet-induced insulin resistance. In addition, decreased activity of liver S6K1 [94], IKK*β*, and JNK1 [95] improves hepatic insulin sensitivity. Reciprocally, S6K1 activation in fat-fed mouse liver or specific activation of hepatic ERK1 by C-reactive protein is linked to steatosis and insulin resistance [96, 97]. These stress kinases are targets of PP2A [98–100], a redox-sensitive protein phosphatase inactivated in an oxidized environment. In addition to enhancing activity of the stress kinases, oxidant-mediated inactivation of PP2A can cause persistent hyper-activation of NF-*κ*B following TNF*α* stimulation [101], which may also have negative consequences on insulin sensitivity. These findings are consistent with evidence that insulin resistance develops as a consequence of aberrant Ser-phosphorylation events within key molecular components of the insulin signalling pathway [82, 102, 103]. However, although the redox sensitivity of Ser/Thr phosphatases is well established, it has not been thoroughly investigated within the context of insulin resistance associated with metabolic disease. In a similar manner to phosphatases, redox modification of kinases may also play a role in ROS-mediated insulin resistance. p38 MAPK is responsible for integrating ROS signals into a phosphorylative cascade [104] and has been shown to mediate palmitate-induced insulin resistance in hepatocytes [105]. The majority of studies did not investigate these pathways in the context of insulin resistance even though kinases involved in insulin signalling (PKB/AKT) are directly regulated by ROS [106]. Relatively few studies have investigated whether these mechanisms hold true for patients with fatty liver disease. Thus, it will be imperative to assess whether altered activity of phosphatase or kinase enzymes, as the result of changing ROS levels, plays a pathogenic a role in worsening insulin sensitivity in NAFLD/NASH.

Due to the intimate involvement of ROS in multiple aspects of insulin signalling, unraveling the timeline and dynamics of ROS production within the context of insulin resistance will help to determine whether or not ROS is a viable target for the treatment of metabolic disease. However, current methods to study ROS in cells and *in vivo* are limiting. The most common method is the redox-sensitive probe H_2_-DCFDA; however it is fairly insensitive at detecting subtle changes in ROS concentration and cannot differentiate ROS production from different cellular compartments. Moreover, endogenous esterases are required to generate the active probe, enzymes that are themselves affected by ROS-inducing treatment and pathological conditions. Probe-based methods allow only snapshots of the oxidative environment, whereas ROS are highly dynamic and transient molecules. Therefore, more sensitive and precise methods are needed to investigate the impact of ROS on cellular signalling if we are to truly understand their role, if any, on the relatively gradual development of insulin resistance in metabolic disease. New advances in electron paramagnetic resonance spectroscopy [107] and amperometry of hydrogen peroxide [108] will hopefully provide a more detailed understanding of the dynamic cellular environment, as both methods are extremely sensitive and can be applied *in vivo*. Single cell fluorescence also allows direct observation of ROS dynamics in different cell organelles [109] and can be applied to *in vitro* systems. Emerging technologies such as proteomic analysis of carbonyl-enriched samples [110–112] and the newly developed NOxICAT technology using a specific redox-sensitive cysteine trap are global approaches toward unraveling pathways impacted by ROS generation [113].

While the molecular mechanisms remain unclear, increased ROS is associated with multiple aspects of the metabolic syndrome including obesity, fatty liver disease, and diabetes, and is thus an attractive therapeutic target to improve insulin sensitivity. Moreover, elevated ROS and oxidative damage are also implicated in inflammation and endoplasmic reticulum stress, additional aggravating factors in the pathogenesis of type 2 diabetes. While inhibiting or reducing ROS seems as appealing strategies to treat metabolic disease, it is clear that ROS are necessary for effective and efficient propagation of intracellular signals, including the insulin signalling cascade. Thus, simple untargeted antioxidant therapy to treat insulin resistance may have multiple effects on signalling pathways independent of preventing oxidative damage. In animal models of metabolic disease, there is incomplete recovery of insulin sensitivity after an antioxidant treatment [57] and only partial prevention of NAFLD progression in rats after N-acetylcysteine administration [114]. In humans, intervention trials restricted to antioxidant supplementation have yielded little, if any, measurable effects on insulin sensitivity in type 2 diabetics [115, 116]. However, in small groups of NASH patients, insulin sensitivity seems to be improved following administration of vitamin E [117]. Mitochondria-targeted therapies such as SS31 or MitoQ may have better chances, as they do not interfere with ROS-potentiated insulin signalling, but prevent rising mitochondrial H_2_O_2_ concentrations. MitoQ administration improves hepatic steatosis, hyperglycemia, and liver damage in high fat-fed ApoE-deficient mice [118] and prevents ROS-induced liver damage in animal models and humans after HCV infection [119], suggesting that detoxification of mitochondrial ROS may have beneficial effects in NASH, which is characterized by both insulin resistance and uncontrolled inflammation. A phase 2 clinical trial was initiated to investigate the benefits of MitoQ for the treatment of fatty liver disease (ID: NCT01167088), but the trial was recently terminated due to poor enrollment.

Mitochondrial dysfunction is a hallmark of NAFLD progression. Subsequent H_2_O_2_ production and emission oxidize redox-sensitive cysteine residues that initially potentiate insulin action, yet potentially lead to insulin resistance via chronic inhibition of phosphatases and aberrant kinase activity. Proteins involved in enhancing mitochondrial function and limiting ROS accumulation, such as PGC-1*α*, Sirt1, and Sirt3, are of interest as specific therapeutic targets to improve insulin resistance observed in NAFLD progression. In addition, there is significant promise in using mitochondrial-targeted antioxidants to prevent or reverse liver damage in NAFLD patients, yet additional preclinical and clinical trials are needed to determine the effectiveness of these strategies. Unravelling the impact of ROS on global hepatocyte signalling and using recent advances in methodology for ROS quantification and localization will help decipher the importance of redox regulation in altered hepatic metabolism to possibly reveal novel, targeted therapeutics for the treatment of NAFLD and NASH.

## Figures and Tables

**Figure 1:**
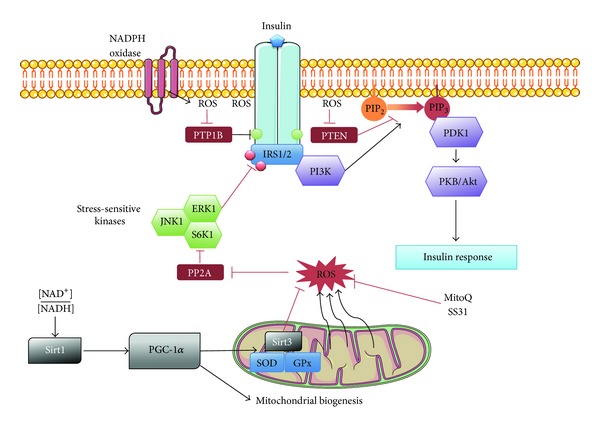
Molecular mechanisms linking ROS, antioxidants, and the insulin signalling pathway. To allow a response to insulin stimulation, ROS are relieving insulin receptor's inhibition by phosphatases such as PTP1B. When the cellular environment shifts to an oxidative one, because of increased mitochondrial respiration and ROS release for example, the stress-sensitive kinases are activated upon redox-sensitive phosphatases inhibition. Theses kinases are inhibiting signal amplification by inhibitory phosphorylation of IRS proteins. Mitochondrial ROS can be regulated by PGC-1*α*-dependent detoxification system (Sirt3, SOD, GPx) or by specific mitochondrial antioxidants (SS31, MitoQ).
